# Randomized phase II clinical trial of ruxolitinib plus simvastatin in COVID19 clinical outcome and cytokine evolution

**DOI:** 10.3389/fimmu.2023.1156603

**Published:** 2023-04-18

**Authors:** Jesus Garcia-Donas, Diego Martínez-Urbistondo, Kyra Velázquez Kennedy, Paula Villares, Arántzazu Barquin, Andrea Dominguez, Juan Francisco Rodriguez-Moreno, Elena Caro, Rafael Suarez del Villar, Estanislao Nistal-Villan, Monica Yagüe, Maria Ortiz, Maria Barba, Sergio Ruiz-Llorente, Miguel Quiralte, Massimiliano Zanin, Cristina Rodríguez, Paloma Navarro, Pedro Berraondo, Rodrigo Madurga

**Affiliations:** ^1^ Gynecological, Genitourinary and Skin Cancer Unit HM CIOCC MADRID (Centro Integral Oncológico Clara Campal), Department of Basic Medical Sciences, Hospital Universitario HM Sanchinarro, HM Hospitales, Institute of Applied Molecular Medicine (IMMA), Facultad de Medicina, Universidad San Pablo CEU, CEU Universities, Madrid, Spain; ^2^ Internal Medicine Service Hospital HM Sanchinarro, Madrid, Spain; ^3^ Hematology Department, Universitario Hospital Ramon y Cajal, Madrid, Spain; ^4^ Microbiology Section, Dpto. CC, Farmacéuticas y de la Salud, Facultad de Farmacia, Universidad San Pablo-CEU, Madrid, Spain; ^5^ Facultad de Medicina, Instituto de Medicina Molecular Aplicada (IMMA), Universidad San Pablo-CEU, Madrid, Spain; ^6^ Laboratory of Innovation in Oncology HM CIOCC MADRID (Centro Integral Oncológico Clara Campal), Department of Basic Medical Sciences, Facultad de Medicina, Hospital Universitario HM Sanchinarro, HM Hospitales, Institute of Applied Molecular Medicine (IMMA), Universidad San Pablo CEU, CEU Universities, Madrid, Spain; ^7^ Clinical Trials Pharmacy, Clara Campal Comprehensive Cancer Center, Hospital Universitario de Sanchinarro, Madrid, Spain; ^8^ Instituto de Física Interdisciplinar y Sistemas Complejos IFISC (CSIC-UIB), Palma de Mallorca, Spain; ^9^ Grupo de Cáncer Endocirno, Centro Nacional de Investigaciones Oncológicas, Madrid, Spain; ^10^ Program of Immunology and Immunotherapy, Cima Universidad de Navarra, Pamplona, Spain; ^11^ Navarra Institute for Health Research (IDISNA), Pamplona, Spain; ^12^ Centro de Investigación Biomédica en Red de Cáncer (CIBERONC), Madrid, Spain; ^13^ Faculty of Experimental Sciences, Universidad Francisco de Vitoria, Madrid, Spain

**Keywords:** COVID19, SARS-Cov-2, cytokine storm, ruxolitinib, simvastatin, clinical trial

## Abstract

**Background:**

Managing the inflammatory response to SARS-Cov-2 could prevent respiratory insufficiency. Cytokine profiles could identify cases at risk of severe disease.

**Methods:**

We designed a randomized phase II clinical trial to determine whether the combination of ruxolitinib (5 mg twice a day for 7 days followed by 10 mg BID for 7 days) plus simvastatin (40 mg once a day for 14 days), could reduce the incidence of respiratory insufficiency in COVID-19. 48 cytokines were correlated with clinical outcome.

**Participants:**

Patients admitted due to COVID-19 infection with mild disease.

**Results:**

Up to 92 were included. Mean age was 64 ± 17, and 28 (30%) were female. 11 (22%) patients in the control arm and 6 (12%) in the experimental arm reached an OSCI grade of 5 or higher (p = 0.29). Unsupervised analysis of cytokines detected two clusters (CL-1 and CL-2). CL-1 presented a higher risk of clinical deterioration vs CL-2 (13 [33%] vs 2 [6%] cases, p = 0.009) and death (5 [11%] vs 0 cases, p = 0.059). Supervised Machine Learning (ML) analysis led to a model that predicted patient deterioration 48h before occurrence with a 85% accuracy.

**Conclusions:**

Ruxolitinib plus simvastatin did not impact the outcome of COVID-19. Cytokine profiling identified patients at risk of severe COVID-19 and predicted clinical deterioration.

**Trial registration:**

https://clinicaltrials.gov/, identifier NCT04348695.

## Background

COVID-19 remains an important health problem worldwide three years after its initial description (1). Though most cases are asymptomatic and vaccines have proven their efficacy, morbidity and mortality rates remain relevant (2–7).

Cytokine storm due to SARS-CoV-2 infection is a critical step in mild and severe disease (8). Although dexamethasone, baricitinib, tofacitinib, and tocilizumab have reported positive results in clinical trials, additional treatment options are required (9, 10).

Janus Kinase (JAK) are essential proteins involved in immune response and could play a role in the hyperinflammatory state in patients with COVID19.

To date, two JAK inhibitors, baricitinib and tofacitinib, have communicated positive results in randomized clinical trials (11, 12).

Ruxolitinib is a selective JAK 1/2 inhibitor that has shown conflicting results in COVID19. Even though several single arm studies have pointed towards a benefit with this drug, a phase III randomized trial has not confirmed such promising activity (13–16).

Simvastatin is a lipid lowering agent with anti-inflammatory properties. Investigators of our group have shown that this compound can block virus internalization mediated by clatrin in cancer models (17). Additionally, simvastatin seems to preclude direct activation of endothelial cells by SARS-CoV-2 Nucleocapsid Protein and inhibits the SARS-CoV-2 main protease (Mpro) (18, 19).

Finally, it has been proposed that patients taking simvastatin could present a more favorable outcome when developing COVID-19 (20, 21).

Thus, we hypothesized that the combination of ruxolitinib plus simvastatin could present a synergistic effect and prevent respiratory and clinical worsening in COVID-19 patients.

As mentioned, cytokine storm seems to be the main cause of severe disease in COVID-19 (8). Several studies have described the immune mediators involved in the development of severe disease, but machine learning algorithms have been required to provide immunotypes that could guide clinical practice (22).

Thus, we used unsupervised clustering and machine learning methodologies to define a minimal number of cytokine determinations that could accurately identify patients at risk of severe COVID and predicted clinical deterioration 48 hours before occurring.

## Objectives

The primary objective of this study was to compare the number of COVID19 patients who progressed to severe disease (defined as grade 5 or more of the OSCI) in the control vs the experimental arm.

Secondary objectives were to compare ICU admission and length of stay, days of hospitalization, and mortality at 28 days, 6 months and 12 months after randomization between study arms. Also, we described the toxicity profile of the combination of ruxolitinib plus simvastatin.

Finally, we aimed to study the evolution of cytokines in plasma along treatment, define a cytokine signature in plasma predictive of COVID-19 outcome and to develop a Machine Learning (ML) algorithm able to predict patient deterioration.

## Methods

We designed a randomized, single-center phase II clinical trial. Patients were allocated in a 1:1 ratio to the control or experimental arm.

Randomization was stratified based on concurrent treatment with statins or strong CYP 340 inhibitors.

### Participants

Eligible cases were adult patients admitted to our institution due to COVID-19 infection. They must have presented with mild disease (defined as grade 3 or 4 in the WHO-Ordinal Scale for Clinical Improvement [WHO-OSCI]) and provided consent (**Supplementary Table 1**).

The study was performed at the Hospital Universitario Sanchinarro, Madrid (Spain).

Treatment in the control arm consisted of standard of care (SOC), including corticosteroids, tocilizumab, heparin and any other therapy considered appropriate by clinical investigators.

The experimental arm consisted of SOC plus the combination of ruxolitinib 5 mg twice a day for 7 days followed by 10 mg BID for 7 days plus Simvastatin 40 mg once a day for 14 days.

Early dose scalation and higher doses of ruxolitinib were permitted on physicians’ discretion. Crossover to the ruxolitinib arm was allowed and continuation of treatment beyond 14 days was also permitted if patients were considered to obtain clinical benefit by the treating physicians.

Patients taking statins before hospital admission, continued with the original medication. Those assigned to the experimental arm received ruxolitinib as referred.

### Outcomes

The primary objective was the percentage of patients progressing from mild (grade 3 or 4 in the OSCI) to severe (grade 5 or more) disease. Secondary objectives included days of hospitalization, days of admission in the intensive care unit (when required), survival at 28 days, 6 months and 12 months after study inclusion and safety profile.

Based on data from our own institution, the likelihood of developing severe respiratory insufficiency was 50% for hospitalized patients (23). A reduction of 28% was expected in the experimental vs the control arm. Thus, 37 patients per group would be required to reach a statistical power (1-β) of 80% with a type I statistical error (α) of 0.05. Assuming a percentage of patient loss of 20%, we aimed to include 94 cases as a whole.

Patients were allocated in a 1:1 ratio to the control or experimental arm.

Cases were stratified based on prior treatment with statins and concomitant treatment with strong CYP 3A4 inhibitors.

Block randomization with a block size of 4 and 6 was used. Blocks were generated by the study statistician who was also responsible for allocating patients in a blinded manner.

### Statistical methods

Quantitative variables are expressed as mean ± standard deviation when normally distributed and median (IQR) otherwise. Normality was tested using the Shapiro test. Categorical variables are expressed as absolute (relative, %) frequencies.

Analysis of the primary objective was reported by treatment arm and performed in both intentions to treat and eligible (“per protocol”) populations. Secondary objectives were only analyzed per protocol.

To compare the main study variable, proportion of patients that reached an OSCI grade of 5 or higher, between control and treated group an homogeneity test was performed with the Chi-squared statistic.

To compare the study variable SpFiO2 worsening below 300 and clinical improvement between study arms, the Chi-squared test was performed. Fisher's exact test was used for the analysis of the categorical variables clinical worsening, SpFiO2 recovery, number of patients requiring ICU admission and mortality rates at 28 days, 6 months and 12 months.

For the continuous variables time to SpFiO2 worsening (< 300), time to clinical improvement, time from randomization to discharge and time to lymphocyte recovery, the Mann-Whitney U test was performed. The t-student test was used for time to SpFiO2 recovery.

### Cytokine analysis

Up to 444 blood samples from 92 patients were collected. 84 (91%) were participants of the Ruxo-Sim trial and 8 extra cases were included providing they met same eligibility criteria. Clinical information and patient demographics were obtained from the electronic medical records, and confidentiality was maintained by assigning each patient a unique identifier. The study protocol was approved by the Ethics Committee of the institution and all patients provided informed consent. Blood collected in EDTA tubes was centrifuged at 1300 x g for 10 minutes, then plasma was aliquoted and stored at −80°C until testing. To avoid additional exposure of healthcare workers to the virus, blood extractions were performed when indicated in routine practice.

### Cytokine characterization

25 µl of neat serum samples from patients were tested using Human Cytokine/Chemokine/Growth Factor Panel A 48 Plex Kit (ref. HCYTA-60K-PX48, Merck KGaA, Darmstadt, Germany) according to the manufacturer’s instructions (see **Supplementary Material** [SM] for details).

### Statistical and machine learning analysis

Hierarchical consensus clustering based on the most variable cytokines resulted in two clusters of patients with distinct cytokine profiles. A logistic regression model was used to classify patients in CL-1 or CL-2 based on a two-cytokine ratio. This model was tested on the 81 patients with a cytokine characterization in the first three days of hospital admission and the association of both groups with demographic and clinical parameters was assessed. (See SM for details on clustering, logistic regression model and validation procedures).

All machine learning (ML) results correspond to two-classes balanced classification problems, tackled through Random Forest models. Input features correspond to cytokine levels, and labels for the two classes are defined according to the patient clinical course, either improving or deteriorating, as measured by laboratory and physical parameters. See SM for details on other ML models, parameter tuning, and validation procedures.

## Results

### Participant flow

**Figure 1:**
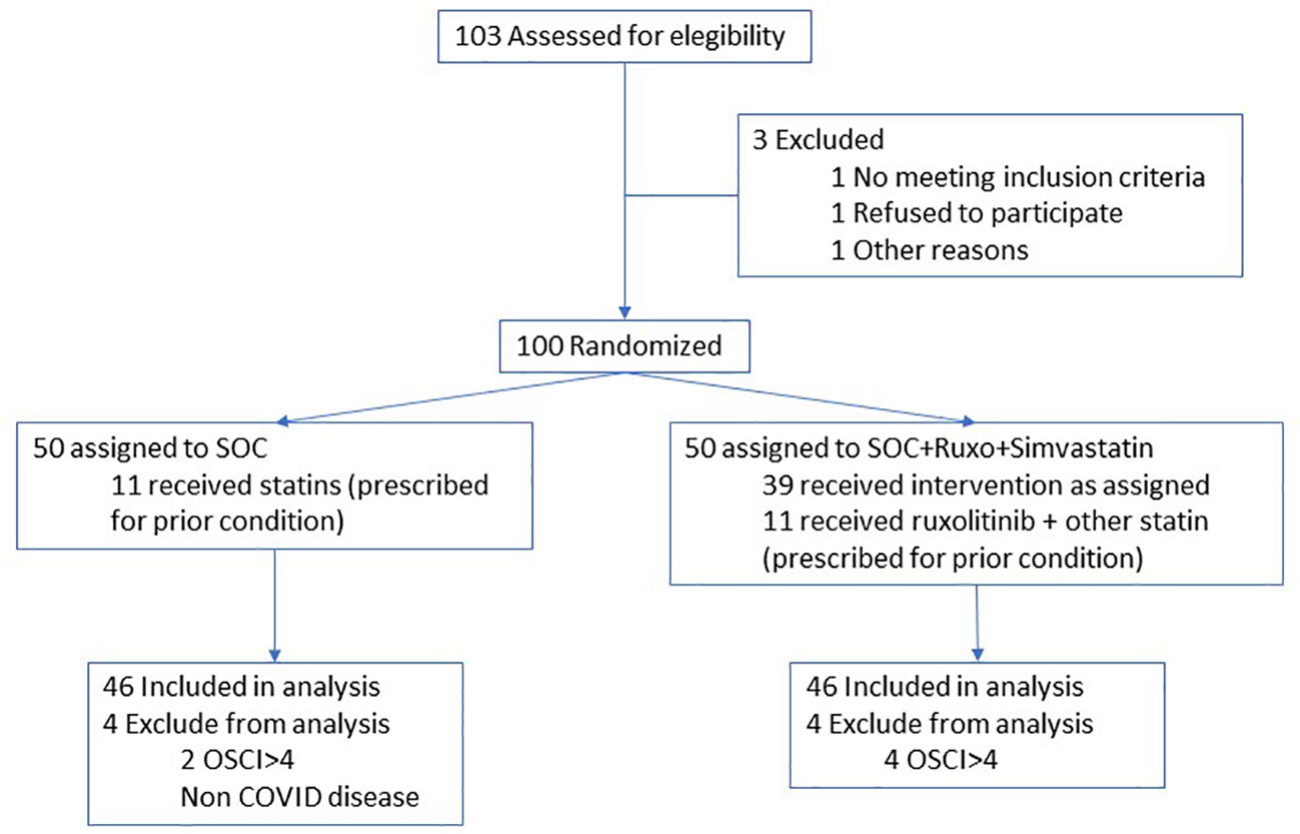
Flow chart of patient inclusion.

100 patients were included in the trial (“intention to treat” population). Eight cases were deemed ineligible after randomization (six presented an OSCI grade greater than four, one was not a COVID patient, and one was included in a competing clinical trial). Thus, the “per protocol” population included 92 patients. (**Figure 1**). Finally, 55 patients received at least one dose of ruxolitinib within the clinical trial and were deemed as the safety population.

Inclusion of cases started in April 2020 and ended in November 2020 after completing the scheduled recruitment. Median follow up was 16 months (range 1-23).

### Baseline data

Mean age was 64 (range 24-98) and 30% were female. Regarding the main clinical prognostic factors in COVID-19: 16% presented diabetes, 13% cardiovascular disease, 12% cancer and 9% obesity. No significant disbalance was observed between study arms regarding these variables (**Table 1**).

**Table 1:** Demographics and basal clinical characteristics.

	Overall population (n = 92)	Control (n = 46)	Ruxolitinib (n = 46)	p-value
**Age** *Mean ± sd*	64 ± 17	67 ± 18	62 ± 16	0.14*
Gender				
Female	28 (30.4%)	15 (32.6%)	13 (28.3%)	0.82**
Male	64 (69.6%)	31 (67.4%)	33 (71.7%)	
Comorbidity				
Hypertension	35 (38.0%)	18 (39.1%)	17 (37%)	1**
*Obesity*	8 ( 8.7%)	3 (6.5%)	5 (10.9%)	0.71***
*Diabetes*	15 (16.3%)	7 (15.2%)	8 (17.4%)	1**
*Cardiovascular*	12 (13.0%)	9 (19.6%)	3 (6.5%)	0.12**
*Trombolembolism*	5 ( 5.4%)	3 (6.5%)	2 (4.3%)	1***
*Cerebrovascular*	1 ( 1.1%)	1 (2.2%)	0 (0%)	1***
*Cancer*	11 (12.0%)	4 (8.7%)	7 (15.2%)	0.52**
*Cancer history*	8 (8.7%)	2 (4.3%)	6 (13.0%)	0.27***
*Others*	75 (81.5%)	38 (82.6%)	37 (80.4%)	1**
Time from event (days)				
Admission	1.00 (0.00 - 1.00)	1.00 (0.00 - 1.00)	1.00 (0.00 - 1.00)	0.92****
Symptom onset *Median (IQR*	9.0 (7.0 - 12.0)	9.0 (6.0 - 11.8)	9.0 (7.0 - 12.0)	0.61****
Blood presure				
Diastolic (*Mean ± sd*)	74 ± 9	73 ± 8	75 ± 11	0.33*
Systolic *Median (IQR)*	123 (117 - 133)	122 (114 - 131)	124 (119 - 133)	0.62****
Temperature (°C)				
*< 37.3*	85 (92.4%)	44 (95.7%)	41 (89.1%)	0.43***
*>= 37.3*	7 ( 7.6%)	2 (4.3%)	5 (10.9%)	
SpFiO2				
<300	3 ( 3.3%)	2 (4.3%)	1 (2.2%)	1***
>300	89 (96.7%)	44 (95.7%)	45 (97.8%)	
OSCI				
3	12 (13%)	5 (10.9%)	7 (15.2%)	0.76**
4	80 (87%)	41 (89.1%)	39 (84.8%)	

OSCI: WHO-Ordinal Scale for Clinical Improvement *t-Student; ** Chi-squared test,*** Fisher test;**** Mann-Whitney U test.

No patient previously treated with a JAK inhibitor was included in the trial and no JAKi, other than ruxolitinib, was administered along the study period.

### Outcomes and analysis

Regarding the primary objective of the study in the intention to treat population, 11 (22%) patients in the control arm and 6 (12%) in the experimental arm reached an OSCI grade of 5 or higher (p = 0.29).

**Figure 2:**
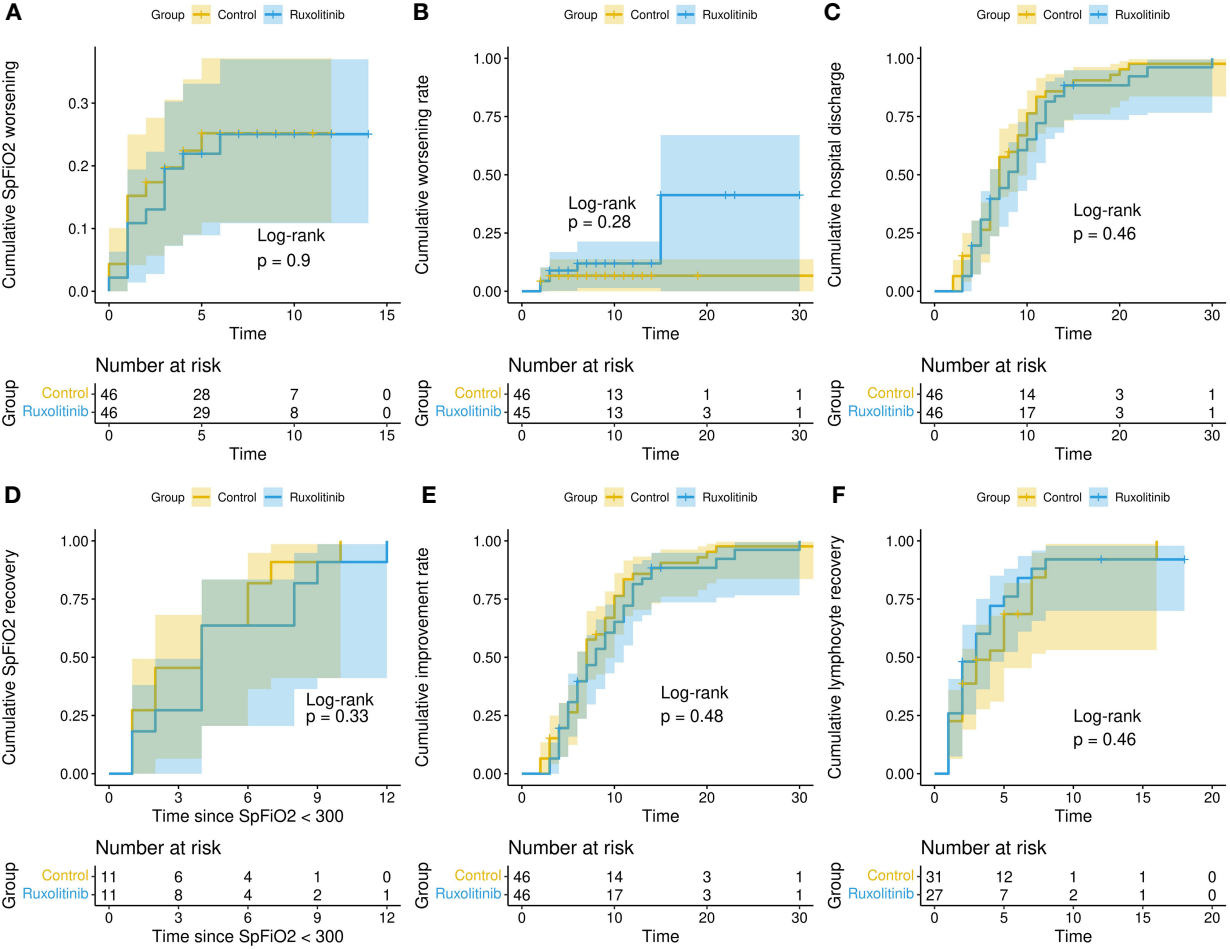
Clinical outcomes. Cumulative distribution for different clinical outcomes (time to event in days): SpFiO2 worsening [< 300] **(A)**; clinical worsening [OSCI > 5] **(B)**, hospital discharge **(C)**; SpFiO2 recovery **(D)**; clinical improvement [reduction of two points on the OSCI scale] **(E)** and recovery from lymphopenia **(F)**.

In the “per protocol” population numbers were smaller (p = 0.0002) with 4 (9%) and 7 (16%) in the control and experimental arms respectively (p = 0.52). Worsening of SpFiO2 was observed in 11 (24%) patients in the control arm and 11 (24%) in the experimental arm (p > 0.99). Median time (days) to SpFiO2 worsening (1 [IQR 1-2.5] vs 2 [IQR 1-3]; p=0.43), time to clinical worsening (2.5 [IQR 2-10.8] vs 3.0 [IQR 2.5-10.5]; p = 0.77) and time from randomization to discharge (7.0 [IQR 5.0 - 10] vs 8.0 [IQR 5.0 - 11.8]; p=0.5) was similar in the control and experimental arms (**Figure 2**).

Three patients required admission to the ICU (one in the control and 2 in the experimental arm) and four died (2 in the control and 2 in the experimental arm).

Additional analysis included the number and percentage of patients with SpFiO2 recovery and clinical recovery, time to clinical improvement, time to SpFiO2 recovery and time to lymphocyte recovery, with no significant difference between groups (**Table 2**).

**Table 2:** Overall population outcome by study arm.

Variable	Overall population (n = 92)	Control (n = 46)	Ruxolitinib (n = 46)	P value
Time to SpFiO2 worsening (< 300) *Median (IQR)*	1.00 (1.00 - 3.00)	1.00 (1.00 - 2.50)	2.00 (1.00 - 3.00)	0.43****
SpFiO2 worsening (< 300)	22 (23.9%)	11 (23.9%)	11 (23.9%)	>0.99**
Time to clinical worsening (*Median [IQR])*	3.0 (2.0 - 10.5)	2.5 (2.0 - 10.8)	3.0 (2.5 - 10.5)	0.77****
Clinical worsening				
*Day 7*	8 (8.7%)	3 (6.5%)	5 (10.9%)	0.71***
*Day 14*	5 (5.4%)	2 (4.3%)	3 (6.5%)	> 0.99 ***
*Day 21*	1 (1.1%)	0 (0.0%)	1 (2.2%)	> 0.99***
*Day 28*	0	0	0	0
Time to SpFiO2 recovery (*Mean ± sd)*	4.6 ± 3.3	4.0 ± 3.0	5.2 ± 3.6	0.41*
SpFiO2 recovery				
*Day 7*	17 (77.3%)	10 (90.9%)	7 (63.6%)	0.31***
*Day 14*	22 (100%)	11 (100.0%)	11 (100.0%)	> 0.99***
Time to clinical improvement *Median (IQR)*	7.0 (5.0 - 11.0)	7.0 (5.0 - 10.0)	7.5 (5.0 - 11.0)	0.55****
Clinical improvement				
*Day 7*	47 (51.1%)	26 (56.5%)	21 (45.7%)	0.4**
*Day 14*	78 (84.8%)	39 (84.8%)	39 (84.8%)	> 0.99**
*Day 21*	83 (90.2%)	43 (93.5%)	40 (87%)	0.48***
*Day 28*	84 (91.3%)	43 (93.5%)	41 (89.1%)	0.71***
Time to cross over *Median (IQR)*	3 (1 - 4)	3 (1 - 4)	–	
Cross over	13 (14.1%)	13 (28.3%)	0 (0%)	–
D28 mortality	3 (3.3%)	1 (2.2%)	2 (4.3%)	> 0.99***
M6 mortality	5 (5.9%)	3 (6.5%)	2 (4.3%)	> 0.99***
M12 mortality	5 (5.9%)	3 (6.5%)	2 (4.3%)	> 0.99***
D28 ICU entry	3 (3.3%)	1 (2.2%)	2 (4.3%)	> 0.99***
Time from randomization to discharge *Median (IQR)*	7.0 (5.0 - 11.0)	7.0 (5.0 - 10.0)	8.0 (5.0 - 11.8)	0.5****
Time to lymphocyte recovery *Median (IQR)*	2.0 (1.0 - 4.0)	3.0 (1.0 - 4.5)	2.0 (1.0 - 4.0)	0.71****
Concomitant medication				
Corticosteroids	71 (77.2%)	35 (76.1%)	36 (78.3%)	> 0.99**
Tocilizumab	27 (29.3%)	13 (28.3%)	14 (30.4%)	> 0.99***
Anticoagulation	89 (96.7%)	44 (95.7%)	45 (97.8%)	> 0.99***

OSCI: WHO-Ordinal Scale for Clinical Improvement *t-Student; ** Chi-squared test, *** Fisher test; **** Mann-Whitney U test.

Up to 13 cases (28%) crossed over to the ruxolitinib treatment arm.

Median time on ruxolitinib treatment was 11 (9 - 12) days for patients allocated to the experimental arm and 11 (8 – 13.8) days for those who were initially assigned to SOC and crossed over to receive ruxolitinib.

All patients who received ruxolitinib reached a dose of 10mg BID or greater.

In the overall ‘per protocol’ population, 30% were on statins before study entry (equally distributed in both arms).

Also, there were no significant differences regarding the administration of corticosteroids (77%), anticoagulants (97%), tocilizumab (29%) or any other drug (**Table 2**; **Supplementary Table 2**).

Regarding toxicity, there were no severe adverse events attributed to the experimental treatment and dose interruptions were not required. All secondary effects were equally distributed between the study arms (**Supplementary Table 3**).

### Cytokine analysis

**Figure 3:**
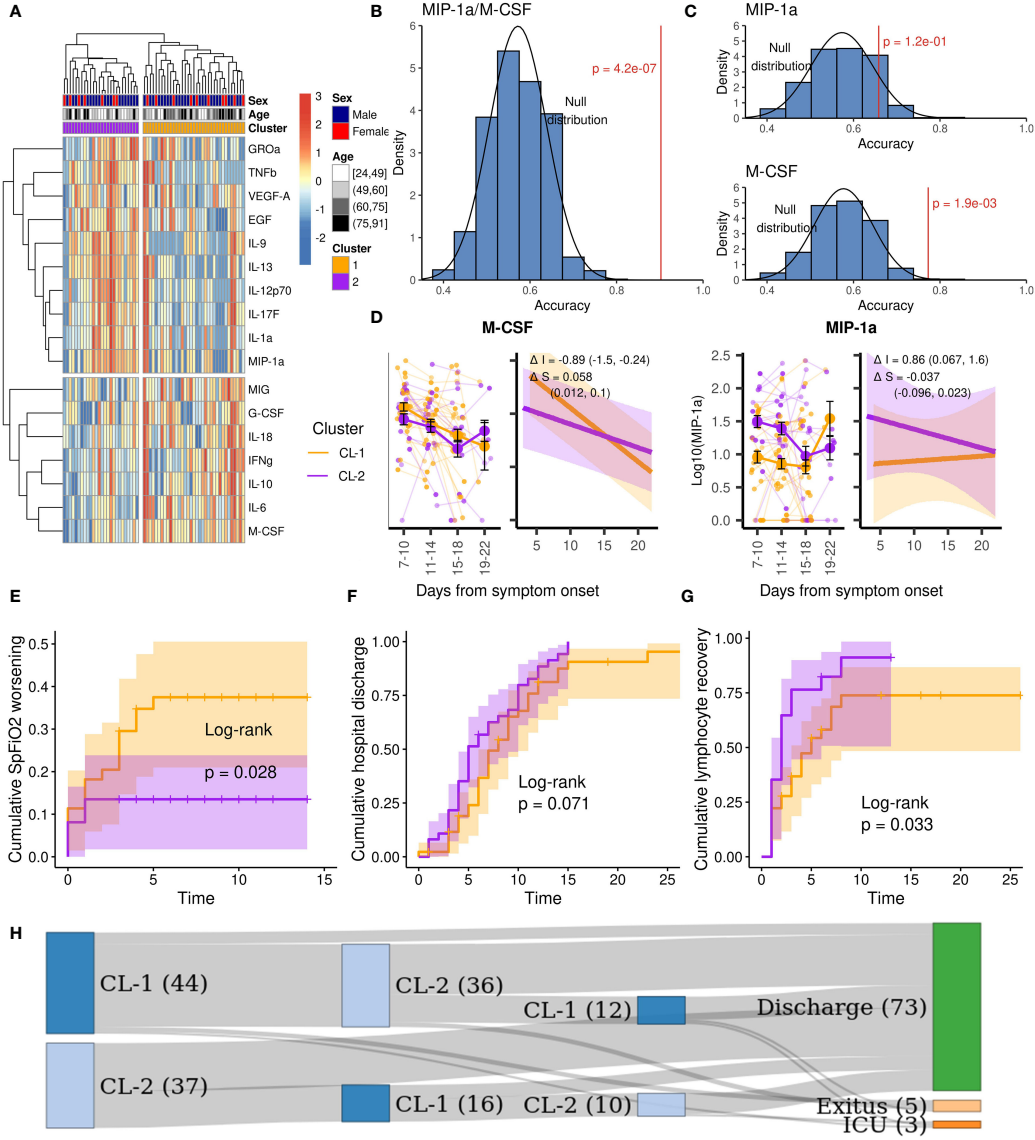
Cytokine clustering and patient’s outcome of the study population. Orange: Cluster 1; purple: cluster 2. **(A)** Clustered heatmap based on the 17 most variable cytokines. **(B)** Accuracy of the MIP-1α/M-CSF ratio for the CL-2 prediction and the null distribution. **(C)** Accuracies of the MIP-1α and M-CSF individually for the CL-2 prediction and the null distributions. **(D)** Expression over time of the most representative cytokines, right plots show the fitted mixed linear models and their variation in fixed intercept and slope effects between clusters. **(E)** Cumulative SpFiO_2_ worsening (< 300) for CL-1 and CL-2. **(F)** Cumulative hospital discharge for CL-1 and CL-2. **(G)** Cumulative lymphopenia recovery for CL-1 and CL-2. **(H)** Cluster progression before outcome (CL-1 is represented in dark blue and CL-2 in light blue).

Consensus clustering of the 61 patients who had a measurement on the first day from hospital admission, based on the 17 most variable cytokines, revealed two clusters in the data (**Figure 3A**; **Supplementary Figure S1**). We used the two-cluster annotation obtained to develop a logistic regression model that classified patients in CL-1 or CL-2 based on two cytokine ratios (see **Supplementary Methods** for details). The ratio MIP-1α/M-CSF reached the best performance with an accuracy of 90 ± 8%, significantly higher than the null distribution (**Figure 3B**), and that obtained by each cytokine individually (**Figure 3C**). Importantly, other cytokines’ ratios obtained similar accuracies **Supplementary Table S4**.

We used the MIP-1α/M-CSF model to classify all 81 patients with cytokine characterization in the first three days from hospital admission. 44 patients were classified as cluster 1 (CL-1) and 37 as cluster 2 (CL-2). **Supplementary Table 5** shows the clinical parameters of both clusters. CL-1 had a higher mean age (68 versus 60, p = 0.041) and higher incidence of hypertension (48% versus 22%, p = 0.027) and cardiovascular disease (16% versus 0%, p = 0.014). CL-1 also had lower levels of neutrophils (median of 3.3 versus 4.9 x103/µL, p = 0.008), lymphocytes (median of 0.77 versus 1.24 x10^3^/µL, p < 0.0001) and platelets (median of 164 versus 229 x10^3^/µL, p = 0.0025) at hospital admission. However, no difference was found in the neutrophil-lymphocyte ratio (NLR, p = 0.18) or in the neutrophil- platelet ratio (NPR, p = 0.77). Nevertheless, 4 (9%) patients from CL-1 died in the first month after hospitalization, while none died in CL-2 (p = 0.12). The difference increased at 12 months, with 5 (11%) deceased patients in CL-1 versus none in CL-2 (p = 0.059). Moreover, a significantly higher cumulative SpFiO_2_ worsening (< 300) was observed in CL-1 (p = 0.028, **Figure 3E**). Additionally, a better prognosis was observed in CL-2 with a trend towards a faster hospital discharge (p = 0.071, **Figure 3F**) and recovery from lymphopenia (p = 0.033, **Figure 3G**) Beyond hospitalization, one patient that remained as CL-1 at discharge required immediate readmission due to clinical deterioration.

No difference regarding cluster distribution was observed between patients on ruxolitinib plus simvastatin or SOC (**Supplementary Table 5**).

To study the expression of individual cytokines in both clusters, we performed linear mixed effects models (**Supplementary Table S6**). As expected, differences in intercept, ΔI, of CL-2 against CL-1 were observed for some cytokines (IL-9, M-CSF, MIP-1α and IL-17A), revealing that initial values are different for those cytokines between clusters. However, differences in slope, ΔS, were only observed for M-CSF (ΔS = 0.058, 95% CI: 0.012, 0.1) as shown in **Figure 3D**, indicating that there were no clear differences in the expression tendency over time between clusters.

When we applied the model to all the measurements performed during the hospital stay of each patient, we found that 8 (18%) of CL-1 did not switch to CL-2. Among those, 1 (13%) was admitted to ICU, 2 (25%) died and 1 (13%) required hospital readmission immediately after discharge due to respiratory deterioration. On the other hand, 21 (57%) of CL-2 did not switch to CL-1, all of them were discharged (**Figure 3H**).

We then analyzed how machine learning models can be trained to classify patients, and what information about the underlying cytokine expression they can yield. For that, a set of models have been trained over balanced sets of patients, according to their future clinical course (see SM for details). The such clinical outcome has been evaluated using three routine clinical parameters: the SpFiO_2_, the D-dimer, and the C-reactive protein. We further considered different time intervals between the two days in which the status of the patient is compared, from one (*δ* = 1, thus assessing whether the patient will improve or worsen the next day) and six (*δ* = 6).

**Figure 4:**
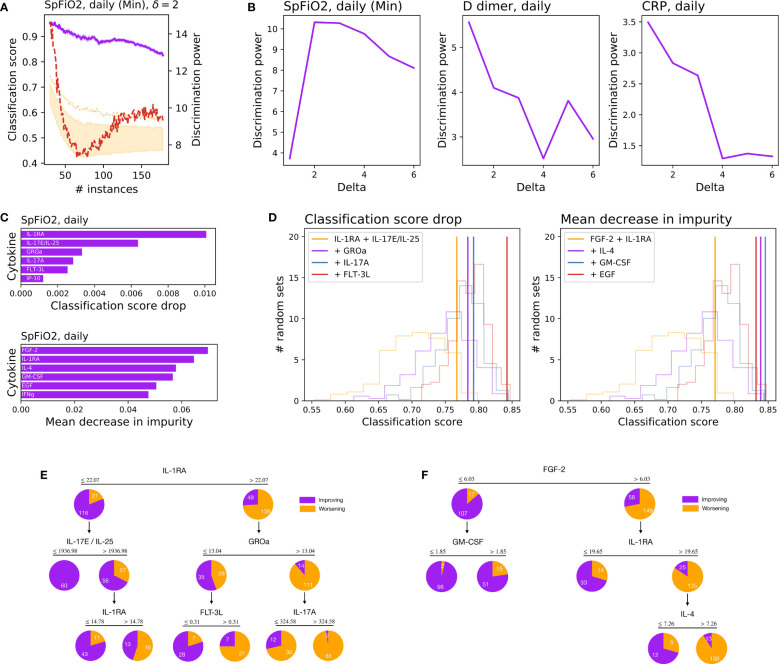
Results of the machine learning analysis and models. **(A)** Classification score (purple line, left Y axis) between two equal-sized groups of most improving and most deteriorating patients, as a function of the number of patients in each group, for a delay of δ=2 days. The orange band and dotted line respectively represent the 10-90 percentile and 99 percentiles of a random classification. The red line (right Y axis) depicts the variations of the discrimination power. **(B)** Variations of the discrimination power, as a function of the number of days, for the three metrics used to assess the status of the patient. **(C)** Ranking of the five most important cytokines for forecasting the status of patients two days ahead, according to the classification score drop (top) and mean decrease in impurity (bottom). **(D)** Classification score obtained by a RF model, using an increased set of cytokines – as ranked in the previous step. Transparent curves represent the probability distributions of the classification score, when models are trained with a random set of features. **(E)** Decision tree for to the five features selected using the classification score drop criterium. **(F)** Decision tree for the four features selected using the mean decrease in impurity criterium.

An example of the resulting classification is presented in **Figure 4A**, as a function of the number of patients composing each group (most improving vs. most deteriorating); results correspond to the SpFiO_2_, for a Random Forest model (see SM for comparisons with other models) and a Leave-One-Out cross-validation. In order to assess the significance of the classification score, a discrimination power has been calculated as the Z-Score between the real result, and an ensemble of results where the true label of patients has been randomly permuted – see red line, right Y axis. As shown, a good classification score of approx. 85% is achieved, corresponding to a Z-Score of above 10.

Panel (b) of **Figure 4** represents the maximum discrimination power obtained for each interval *δ*, for the three metrics considered – full results are included in SM. While the best results for the SpFiO_2_ are obtained for a time interval of two days, the maximum for D-dimer and CRP corresponds to one day. The cytokines involved in the classification of patients (for *δ* = 2, SpFiO_2_, and 165 samples in each group) have been ranked further using two complementary methods: the drop in the classification score and the mean decrease in impurity. Results, depicted in **Figure 4C**, suggest that two sets of complementary cytokines can be defined, each of them with similar classification power (see **Figure 4D**). These two groups of cytokines have finally been used to train two decision trees – see **Figures 4E, F**. Even a simple analysis of the levels of IL-1RA and FGF-2 can yield a good prediction of the future outcome of patients, with high values being associated with a worse prognosis. To illustrate, 159 of the 171 determinations having IL-1RA >= 22.07 and FGF-2 >= 6.03, i.e. the 93.0%, predicted clinical deterioration in a two-day window (Classification scores, **Figures S3**-**6**; features selection **Figure S7**).

## Discussion

We present the results of a single-center randomized phase II clinical trial with the combination of ruxolitinib and simvastatin for the treatment of COVID19. Up to 100 hospitalized patients were included and allocated in a 1:1 ratio to SOC or the experimental arm. Treatment was well tolerated but no differences were found regarding the primary objective, number of cases progressing to grade 5 or higher of the OSCI. Thus, the administration of ruxolitinib and simvastatin did not impact the outcome of COVID19.

A comprehensive cytokine profiling was also completed. Up to 48 cytokines were determined in peripheral blood at baseline and during hospital stay. The ratio MIP-1α/M-CSF reliably classified cases in poor (cluster 1 [CL-1]) and good (cluster 2 [CL-2]) prognosis groups. CL-1 presented a higher likelihood of worsening SpFiO_2_ and death. Most CL-1 cases transitioned to CL-2 before being discharged while those who did not, presented more deaths, ICU admission or required hospital readmission after discharge.

Additionally, a decision tree was designed by ML algorithms that accurately predicted patient deterioration 48 hours before occurring. Both tools, baseline immunoprofiling, and decision tree, could greatly help in the early recognition of patients at risk of severe disease and the precise moment where more intensive care is required.

Although vaccines have dramatically changed the outcomes of patients, the pandemic is far from being controlled since new variants are emerging (24).

Hyperinflammation is an over exaggerated reaction of the immune system due to the SARS-Cov-2 infection that can lead to multiorgan dysfunction and particularly to a respiratory distress syndrome (RDS). This complication is responsible for most deaths and a major cause of long-term effects caused by COVID (8).

In this setting, dexamethasone has demonstrated an impact on overall mortality in a randomized phase III clinical trial and has been adopted as standard of care (9). On the other hand, tocilizumab, an interleukin-6 receptor antagonist, reached more conflicting results and is commonly administered to patients that do not respond to initial therapies (10, 25).

JAK inhibitors have also been proposed as potential alternatives in precluding hyperinflammation. Baricitinib and tofacitinib have communicated positive results in phase III randomized trials (named ACTT-2 and STOP-COVID respectively), while ruxolitinib has not (RUXCOVID trial).

RUXCOVID was an international trial of ruxolitinib plus standard of care versus placebo plus standard of care in patients with COVID-19. Patients who were hospitalised but not on mechanical ventilation were randomly assigned (2:1) to oral ruxolitinib 5 mg twice per day or placebo for 14 days. 432 were included in the study. The primary objective (a composite of death, respiratory failure, or ICU care) was not met.

The RUXCOVID-DEVENT trial compared the activity of two doses of ruxolitinib (5 mg or 15mg twice a day) versus placebo in more than 200 cases hospitalized with severe acute respiratory syndrome due coronavirus 2 (26). However, no significant difference in mortality was observed.

Finally, a single blinded phase II randomized trial that included 43 cases allocated 1:1 to ruxolitinib or placebo did not show significant differences either (27).

Though our study did not reach positive results, some limitations must be discussed.

First, our work represents a smaller population compared to prior trials (1067 in ACTT-2, 287 in STOP-COVID and 432 in RUXCOVID) and was not placebo controlled. Thus, our design could be underpowered to identify statistically significant differences.

Secondly, we decided to focus on moderate disease excluding patients with an OSCI grade of 5 or higher. This is an important factor since corticosteroids, IL-6 antagonists and JAKi have shown a greater clinical impact in severely ill patients.

Thirdly, any concurrent treatment was allowed at investigators discretion in Ruxo-Sim. Thus, 77% of patients received corticosteroids and 29% tocilizumab. In comparison, only 22% received corticosteroids in the ACT-2 trail and Il-6 antagonists were not allowed in either ACTT-2 or STOP-COVID. Thus, a redundant activity between different anti-inflammatory drugs is plausible.

Additionally, the deterioration rate (less than 15% [11 out of 92]) and overall mortality (5% [4 out of 92]) observed in our study was significantly better than expected in a first wave of COVID-19. This could suggest an effectiveness of the control arm more than a poor performance of the experimental arm.

Finally, though prior treatment with statins was a stratification factor, the presence of a component of the combination in the control arm itself determines a significant bias.

Regarding the cytokine profiling analysis, several authors have classified COVID-19 patients in prognostic groups based on immunoprofiles defined with a variety of methods from multiplex cytokines to flow or mass cytometry, scRNAseq, and machine learning (22, 28–31).

As a whole, these studies confirm the interest in characterizing the immune response to predict the clinical course of COVID-19. Unfortunately, most results are far from a clinical application because of the high complexity of the methods, that are not available in most centers.

In contrast, our strategy, taking into account not only the higher concentrations of predefined cytokines but also assessing the difference between up- and down-regulated cytokines, was able to reduce the number of determinations required for a reliable prediction. Since several pairs of cytokines reached an optimal threshold of accuracy, most clinical centers could use determinations already available at their institutions to implement this model. This is key in a global threat like COVID-19.

Interestingly, there was no difference regarding cluster distribution between patients on ruxolitinib plus simvastatin or SOC. This result points to the notion than the extensive use of standard of care treatments, capable of altering the inflammatory state, could preclude the formal evaluation of the real therapeutic role of the experimental agents in our study.

Regarding the decision tree, it has proven to accurately predict the appearance of biochemical alterations 24 hours and clinical deterioration 48 hours before occurring.

This suggests a progressive deterioration of conditions, in which an alteration of the cytokine profile manifests after one day in an inflammatory process, with altered D-dimer and CRP, but with no impact yet in the blood oxygenation, which is only affected after two days.

In summary, though the combination of ruxolitinib plus simvastatin did not show to be superior to standard of care in our study, design limitations like allowing statins in both arms or the better performance than expected of the control arm preclude a definitive conclusion.

Several cytokines, assessed in pairs, classified COVID-19 patients into high- and low-risk groups. A decision tree predicted clinical deterioration 48 hours before occurring. Both tools could help to better tailor treatment for COVID-19 patients.

## Data availability statement

The original contributions presented in the study are included in the article/**Supplementary Material**. Further inquiries can be directed to the corresponding author.

## Ethics statement

The studies involving human participants were reviewed and approved by the ethics committee at HM Hospitales and the Spanish National Agency for Drugs and Health Products (AEMPS). The patients/participants provided their written informed consent to participate in this study.

## Author contributions

Conception and design of study: JG-D, DM-U, KK, PV, AB, EN-V, CR, PN, PB, RM. Acquisition of data (laboratory or clinical): JG-D, DMU, KK, PV, AB, AD, JR-M, EC, RV, EN-V, MY, MO, MB, SR-L, MQ, MZ, CR, PN, PB, RM. Data analysis and/or interpretation: JG-D, DM-U, KK, PV, AB, EN-V, MZ, CR, PN, PB, RM. Drafting of manuscript and/or critical revision: JG-D, MZ, CR, PN, PB, RM. Approval of final version of manuscript: JG-D, DM-U, KK, PV, AB, AD, JR-M, EC, RV, EN-V, MY, MO, MB, SR-L, MQ, MZ, CR, PN, PB, RM. All authors contributed to the article and approved the submitted version.
