# Supplementary material for: Randomized phase II clinical trial of ruxolitinib plus simvastatin in COVID19 clinical outcome and cytokine evolution
Source: Front Immunol. 2023 Apr 18;14:1156603. doi: 10.3389/fimmu.2023.1156603 (PMC10151807; doi:10.3389/fimmu.2023.1156603)
Supplement: Supplementary file 1 [file DataSheet_1.pdf]

SUPPLEMENTARY MATERIAL

Supplementary Table 1. WHO-Ordinal Scale for Clinical Improvement (OSCI)

Patient Scale	Descriptor	Score
**Uninfected**	No clinical or virological evidence of infection	0
**Ambulatory**	No limitation of activities	1
	Limitation of activities	2
**Hospitalized Mild Disease**	Hospitalized, no oxygen therapy	3
	Oxygen by mask or nasal prongs	4
**Hospitalized Severe Disease**	Non-invasive ventilation or high-flow oxygen	5
	Intubation and mechanical ventilation	6
	Ventilation + additional organ support - pressors, RRT ECMO	7
**Dead**	Death	8

Supplementary Table 2. Concomitant medication

. *t-Student; ** Chi-squared test,*** Fisher test;**** Mann-Whitney U test

**Variable**	**Overall population (n = 92)**	**Control (n = 46)**	**Ruxolitinib (n = 46)**	**p value**
**TREATMENT**				
INHALED MEDICATION	44 (47.8%)	22 (47.8%)	22 (47.8%)	1
ANALGESICS	41 (44.6%)	16 (34.8%)	25 (54.3%)	0.0933
ANSIOLITIC MEDICATION	31 (33.7%)	12 (26.1%)	19 (41.3%)	0.186
ANTIPLATELET	9 (9.8%)	7 (15.2%)	2 (4.3%)	0.158
ANTIBIOTIC	88 (95.7%)	43 (93.5%)	45 (97.8%)	0.617
ANTICOAGULANT	89 (96.7%)	44 (95.7%)	45 (97.8%)	1
ANTIDEPRESSANTS	10 (10.9%)	2 (4.3%)	8 (17.4%)	0.0901
ANTIDIABETICS	49 (53.3%)	24 (52.2%)	25 (54.3%)	1
ANTIEMETICS	15 (16.3%)	9 (19.6%)	6 (13%)	0.572
ANTIHYPERTENSIVE DRUGS	34 (37%)	17 (37%)	17 (37%)	1
ANTIPSYCHOTICS DRUGS	13 (14.1%)	7 (15.2%)	6 (13%)	1
ANTIVIRAL	15 (16.3%)	5 (10.9%)	10 (21.7%)	0.259
CHLOROQUINE	29 (31.5%)	13 (28.3%)	16 (34.8%)	0.654
CORTICOSTEROIDS	71 (77.2%)	35 (76.1%)	36 (78.3%)	1

LIPID LOWERING AGENTS	26 (28.3%)	14 (30.4%)	12 (26.1%)	0.817
THYROID MEDICATION	7 (7.6%)	3 (6.5%)	4 (8.7%)	1
PPI	76 (82.6%)	39 (84.8%)	37 (80.4%)	0.783
MUCOLYTIC	16 (17.4%)	6 (13%)	10 (21.7%)	0.409
TOCILIZUMAB	27 (29.3%)	13 (28.3%)	14 (30.4%)	1

Supplementary Table 3. Secondary effects all grades

ADVERSE EVENT	Total (n=92)	Control (n=46)	Experimental (n=46)
**Respiratory disorders**			
Pneumonia	92 (100%)	46 (100%)	46 (100%)
Dyspnea	28 (30%)	12 (26%)	16 (35%)
Cough	33 (36%)	14 (30%)	19 (41%)
**Vascular disorders**			
Thrombus	17 (18%)	8 (17%)	9 (20%)
Hypertension	1 (1%)	1 (2%)	0
**General disorders**			
Fever	20 (22%)	11 (24%)	9 (20%)
Fatigue	15 (16%)	9 (20%)	6 (13%)
Edema	1 (1%)	1 (2%)	0
**Gastrointestinal disorders**			
Vomiting	4 (4%)	3 (7%)	1 (2%)
Diarrhea	12 (13%)	5 (11%)	7 (15%)
Constipation	2 (2%)	1 (2%)	1 (2%)
Mucositis	1 (1%)	1 (2%)	0
**Skin disorders**			
Rash	2 (2%)	1 (2%)	1 (2%)
Pruritus	2 (2%)	1 (2%)	1 (2%)
**Psychiatric disorders**			
Insomnia	1 (1%)	0	1 (2%)
**Blood disorders**			
Anemia	10 (11%)	3 (7%)	7 (15%)
Neutropenia	5 (5%)	1 (2%)	4 (9%)
Lymphopenia	20 (22%)	9 (20%)	11 (24%)
Thrombocytopenia	17 (18%)	8 (17%)	9 (20%)
**Metabolism disorders**			
Hyponatremia	8 (9%)	1 (2%)	7 (15%)

Hypocalcemia	1 (1%)	1 (2%)	0
Hyperkalemia	2 (2%)	1 (2%)	1 (2%)
Hypokalemia	2 (2%)	2 (4%)	0
Hypertransaminasemia	21 (23%)	12 (26%)	9 (20%)
Hyperglycemia	1 (1%)	1 (2%)	0
**Infections**			
Otitis	1 (1%)	0	1 (2%)

Supplementary Table 4. Cytokine ratios (submitted as excel format)

Supplementary table 5. Clinical characteristics of the whole cohort (and divided by clusters) of patients included in cytokine analysis.

Clinical characteristics	All (n = 92)	First determination before day 3 (n = 81)	CL-1 (n = 44)	CL-2 (n = 37)	P Value
Participant in Ruxo-Sim trial	84 (91%)	73 (90%)	39 (89%)	34 (92%)	.72
Control	41 (45%)	35 (43%)	18 (41%)	17 (46%)	.93
Treatment	43 (47%)	38 (47%)	21 (48%)	17 (46%)	
Age, mean (SD), years	66 (17)	65 (17)	68 (15)	60 (18)	.041
Sex (%)					
Female	30 (33)	26 (32)	13 (30)	13 (35)	.77
Male	62 (67)	55 (68)	31 (71)	24 (65)	
Comorbidities (%)					
Hypertension	36 (39)	29 (36)	21 (48)	8 (22)	.027
Cardiovascular disease	11 (12)	7 (9)	7 (16)	0 (0)	.014
Obesity	8 (9)	7 (9)	6 (14)	1 (3)	.12
Diabetes	14 (15)	13 (16)	6 (14)	7 (19)	.73
Cancer	14 (15)	8 (10)	5 (11)	3 (9)	.72
Sp/Fi O_2 at admission, median (IQR)	352 (318 – 369)	353 (322 - 374)	354 (338 - 377)	352 (316 - 368)	.49
Neutrophils, median (IQR), x10^3/µL	4.5 (3.0 – 6.7)	4.2 (2.9 - 6.7)	3.3 (2.4 - 6.1)	4.9 (3.8 - 7.6)	.008
Lymphocytes, median (IQR), x10^3/µL	.93 (.71 – 1.40)	.94 (.71 - 1.38)	.77 (.48 - .98)	1.24 (.93 - 1.69)	< .0001
Platelets, median (IQR), x10^3/µL	191 (150 – 270)	188 (144 - 256)	164 (132 - 218)	229 (178 - 330)	.0025
NPR, median (IQR)	2.2 (1.5 – 3.3)	2.3 (1.5 - 3.4)	2.1 (1.4 - 3.8)	2.4 (1.5 - 3.3)	.77
NLR, median (IQR)	4.6 (2.6 – 7.8)	4.4 (2.5 - 7.9)	4.8 (3.4 - 7.9)	3.7 (2.2 - 7.3)	.18

D-dimer, median (IQR), ng/mL	590 (409 – 1022)	550 (392 - 1015)	530 (389 - 1274)	590 (399 - 992)	.78
CRP, median (IQR), mg/L	60 (33 – 103)	59 (33 - 95)	59 (45 - 110)	56 (32 - 87)	.28
Outcomes (%)					
Sp/Fi O_2 < 300	22 (26)	15 (20)	13 (33)	2 (6)	.0092
Thrombus	16 (17)	12 (15)	6 (14)	6 (16)	.99
Lymphopenia	60 (65)	52 (64)	37 (84)	15 (41)	.00012
ICU (28 days)	4 (4)	3 (4)	2 (5)	1 (3)	1
Exitus (28 days)	4 (4)	4 (5)	4 (9)	0 (0)	.12
Exitus (6 months)	7 (8)	4 (5)	4 (9)	0 (0)	.12
Exitus (12 months)	8 (9)	5 (6)	5 (11)	0 (0)	.059
Treatment					
Chloroquine	26 (28)	15 (19)	9 (21)	6 (16)	.84
Antiviral	20 (22)	18 (22)	10 (23)	8 (22)	1
Heparin	90 (98)	79 (98)	42 (96)	37 (100)	.5
Corticosteroids	74 (80)	67 (83)	37 (84)	30 (81)	.95
Tocilizumab	31 (34)	26 (32)	19 (43)	7 (19)	.036

Supplementary Table 6. Linear mixed effects models (submitted as excel format)

Supplementary Table 7. Individual cytokine analysis (submitted as excel format)

Cytokine characterization

Briefly, 25 µl of assay buffer, 25µl of sample or standard, 25 µl of matrix solution, and 25 µl of mixed beads were added to each well of a 96 well plate and incubated overnight on an orbital shaker at 4°C. After washing, 25 µl of detection antibody were added to each well and incubated on an orbital shaker for one hour at room temperature. Following incubation, 25 µl of streptavidin-phycoerythrin was added to each well and incubated on an orbital shaker for 30 minutes at room temperature. The plate was washed and Mean fluorescence intensity (MFI) per bead was acquired in a Magpix instrument with xPONENT software. Concentrations in samples were calculated by interpolation from standard curves adjusted to 4 parameter or 5 parameter logistic curves.

Cytokine panel

The analyzed cytokines/chemokines/growth factors were (chemokine systematic name is shown in parenthesis): sCD40L, EGF, Eotaxin (CCL11), FGF-2, Flt-3 ligand, Fractalkine (CX3CL1), G-CSF, GM-CSF, GROα (CXCL1), IFNα2, IFNγ, IL-1α, IL-1β, IL-1ra, IL-2, IL-3, IL-4, IL-5, IL-6, IL-7, IL-8, IL-9, IL-10, IL-12 (p40), IL-12 (p70), IL-13, IL-15, IL-17A, IL-17E/IL-25, IL-17F, IL-18, IL-22, IL-27, IP-10 (CXCL10), MCP-1 (CCL2), MCP-3, M-CSF, MDC (CCL22), MIG (CXCL9), MIP-1α (CCL3), MIP-1β (CCL4), PDGF-AA, PDGF-AB/BB, TGFα, TNFα, TNFβ, and VEGF-A.

Routine laboratory tests

Routine laboratory parameters (including LDH, ferritin, D-dimer, and CRP) were determined following standard procedures at the Department of Clinical Chemistry, Hospital HM Sanchinarro.

Cytokine statistical analysis

Continuous variables were tested for normality using Shapiro test. Normally distributed variables were summarized as mean (standard deviation), and means were compared using t test. Non-normally distributed variables were summarized as median (IQR), and means were compared using Mann-Whitney U test. Categorical variables were summarized as absolute frequency (%) and analyzed using chi-squared test or Fisher test when needed. All the statistical analysis were performed using R (version 4.1.1). We considered a statistical threshold of 0.05.

Consensus clustering

We used all the patients with cytokine characterization on the first day of hospital admission (n = 61) for discovery. Cytokines' concentrations were log transformed adding one unit to all the values. Cytokines with median absolute deviation (MAD) higher than 1 across all patients were filtered and used for a consensus clustering using ConsensusClusterPlus R package.[20] A maximum of 6 clusters were fixed and the algorithm was trained over 10,000 repetitions using 80% of the samples. Average hierarchical clustering with Pearson distance was used. The 61 patients clustered in two different clusters as shown in supplementary figure 1.

Logistic regression model

To classify new samples in one of the two found clusters we developed a logistic regression classification model. The 61 samples that were clustered were annotated with the corresponding cluster. Then, the association of each cytokine with cluster 2 was achieved by logistic regression ($p < 0.2$). A total of 240 cytokine ratios were analyzed dividing one of the 20 positively associated cytokines by one of the 12 negatively associated cytokines (supplementary table S7). To avoid results obtained by chance, cross validation with 1,000 iterations and taking 80% of the training cohort was applied. Additionally, a null distribution was obtained by randomly assigning cluster 1 and 2 to each sample, keeping the original proportions of both clusters in the cohort, and repeating the classification algorithm 1,000 times.

Linear mixed effect models

Cytokine production over time was assessed using linear mixed effects models to fit log10 cytokine concentration against days from symptom onset with the lme4 package. Models were built to study the variation in intercept and slope by the characteristic studied (treatment: tocilizumab, corticosteroids or statins; and adverse events: $SpFiO_2$ < 300 or D-dimer > 1,000 ng/mL) while considering random effects by patient in both intercept and slope.Most patients received corticosteroids at some point during their inhospital stay. Different kinds of corticosteroids and dosages were used following physicians' criteria. We transformed doses to their prednisone equivalent dosing and

set a cumulative dosage of 100 mg as threshold to consider a patient in the corticosteroid treated group.

Machine Learning analysis

All machine learning (ML) models have been constructed around a two-class problem, respectively composed of a given number of most improving and most deteriorating patients. The two classes are always composed of the same number of instances, thus ensuring a balanced data set. Note that, if all patients were included at the same time, the result would be an unbalanced sets, and hence results more difficult to interpret. Patient clinical course is predicted using cytokine levels as input features and is assessed through changes in three parameters: SpO_2/Fi ratio, D-dimer and CRP. Several classical ML models have been considered, with the one yielding the best results being the Random Forest (RF) (see Fig. S2) for details and models' parameter tuning).[21] All models have been tested using a Leave-One-Out Cross-Validation, and the classification score has been calculated as the mean accuracy; note that other metrics, like precision or F_1-score, are redundant due to the use of balanced data sets.[22]

Results are reported as the progression of the classification score as a function of the number of patients included in each group – thus of the magnitude of the patient progression. In other words, when few patients are considered, these correspond to the largest variation in clinical course. To compensate for the stochastic nature of RF models, the score corresponds to the average of 1,000 independent realizations. The discrimination power is further evaluated by calculating a Z-Score, between the obtained score, and the score yielded by a set of 1,000 classifications in which labels are randomly shuffled. In the best performing models, the importance of features (i.e. cytokines) is estimated through the average drop in classification score, and through the mean decrease in impurity within the RF model.[23] Finally, explainable models have been extracted in the most representative cases, by training Decision Tree models using the cytokines previously selected.[24]

All ML analyses have been performed in Python 3.8, using the SciKit-Learn library.[25]

Consensus clustering

Figure S1. Identification of two cytokine clusters. (a) Consensus clustering matrix of 56 samples for k = 2 to k = 5. (b) Consensus clustering CDF for k = 2 to k = 6. (c) Cluster consensus for k = 2 to k = 6.

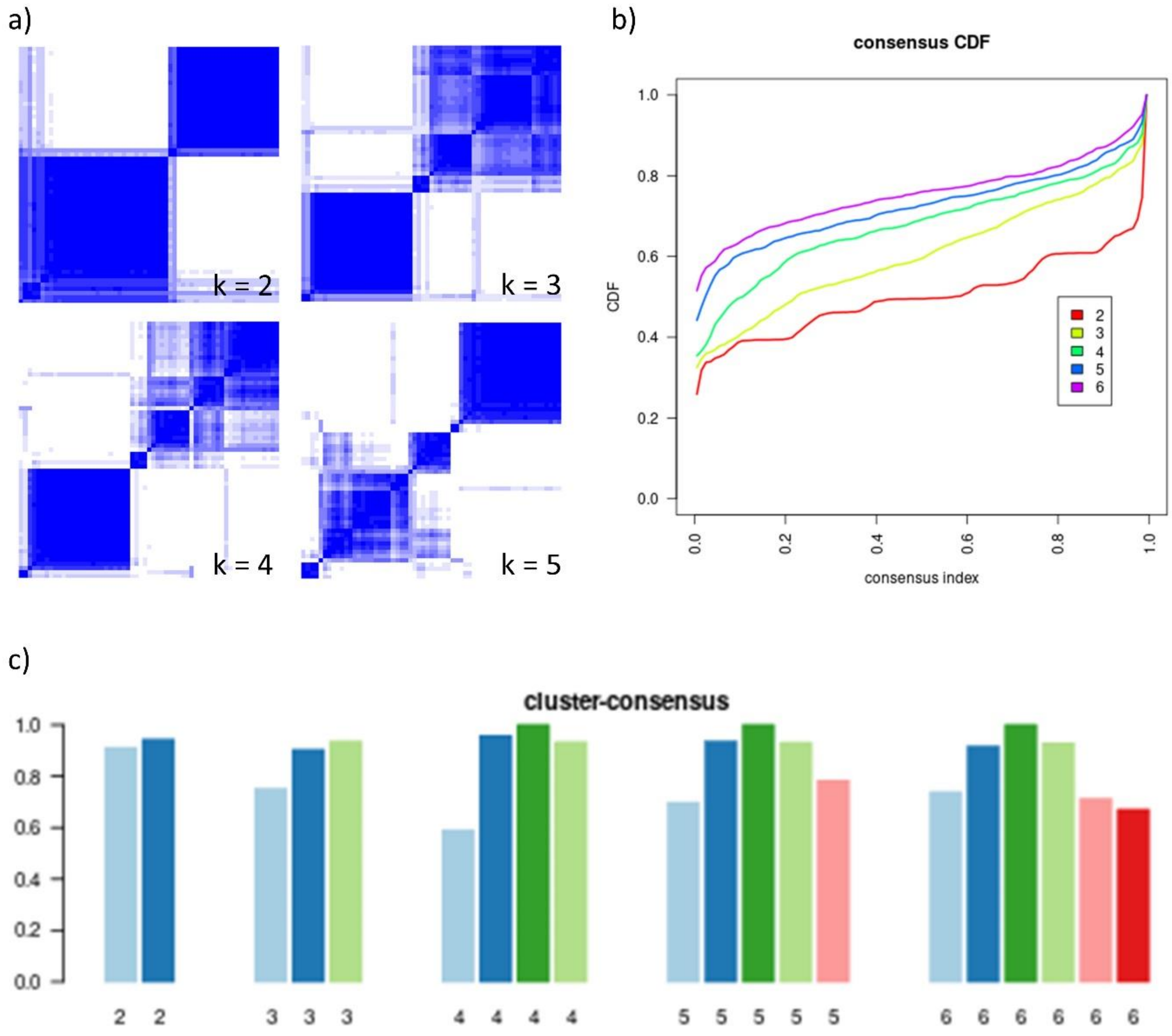

Model comparison and tuning

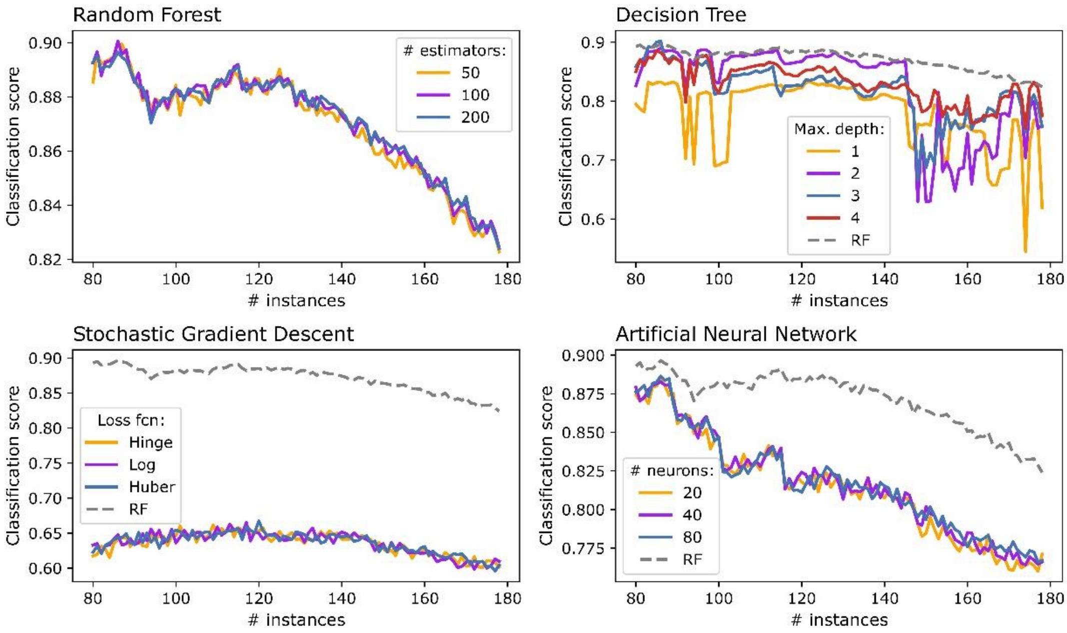

Figure S2. Model comparison and tuning. The panels show the classification score, as a function of the number of instances included in the analysis, using four different classification models. From left to right, top to bottom, these are Random Forests (with lines corresponding to different numbers of estimators); Decision Trees (maximum depth of the model); Stochastic Gradient Descent (loss functions); and two-layers Artificial Neural Networks (number of neurons in each layer). The dashed grey lines represent the classification score of the best model, i.e. Random Forests with 200 estimators.

Classification score

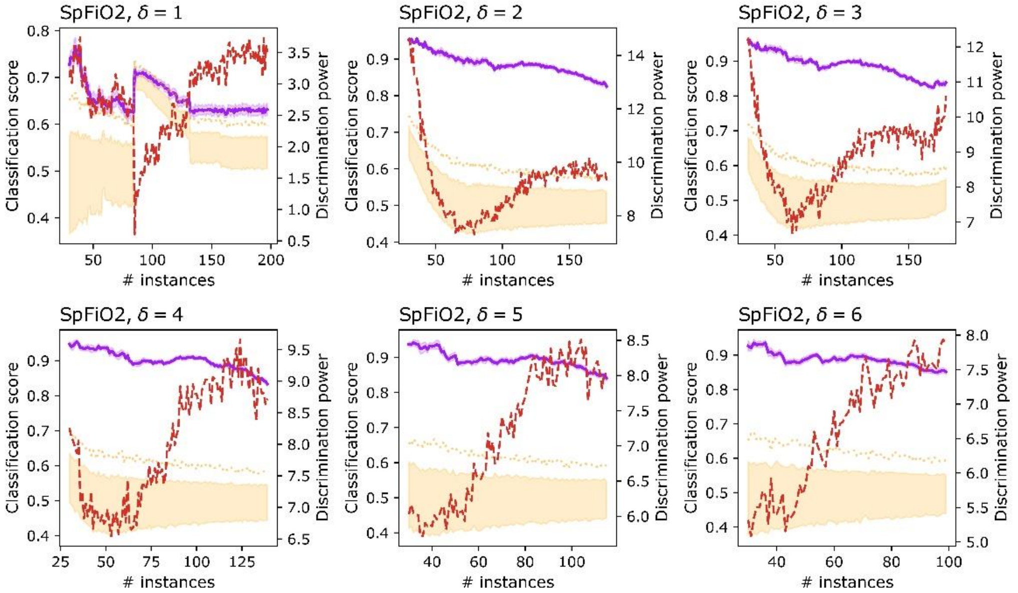

Figure S3. Classification score. Patients improving vs. deteriorating, as measured by the $SpFiO_2$, as a function of the number of instances. Each panel represents a different number of days between the cytokine measurement and the assessment of the condition (δ). The purple lines represent to the classification score (left Y axes), the red lines, the discrimination power (right Y axes).

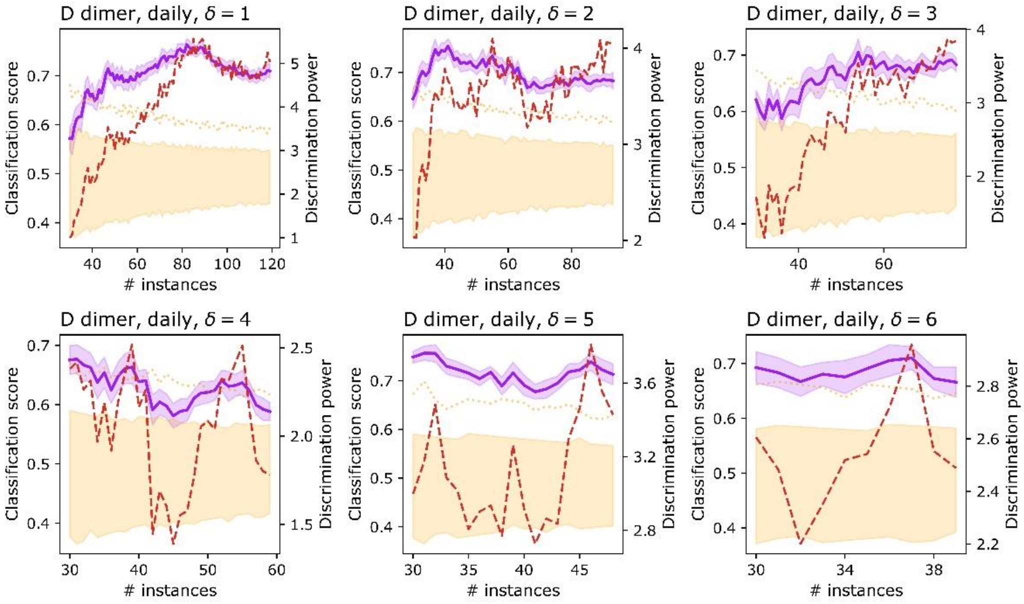

Figure S4. Classification score. Patients improving vs. deteriorating, as measured by the D dimer, as a function of the number of instances. Each panel represents a different number of days between the cytokine measurement and the assessment of the condition (δ). The purple lines represent the classification score (left Y axes), the red lines, the discrimination power (right Y axes).

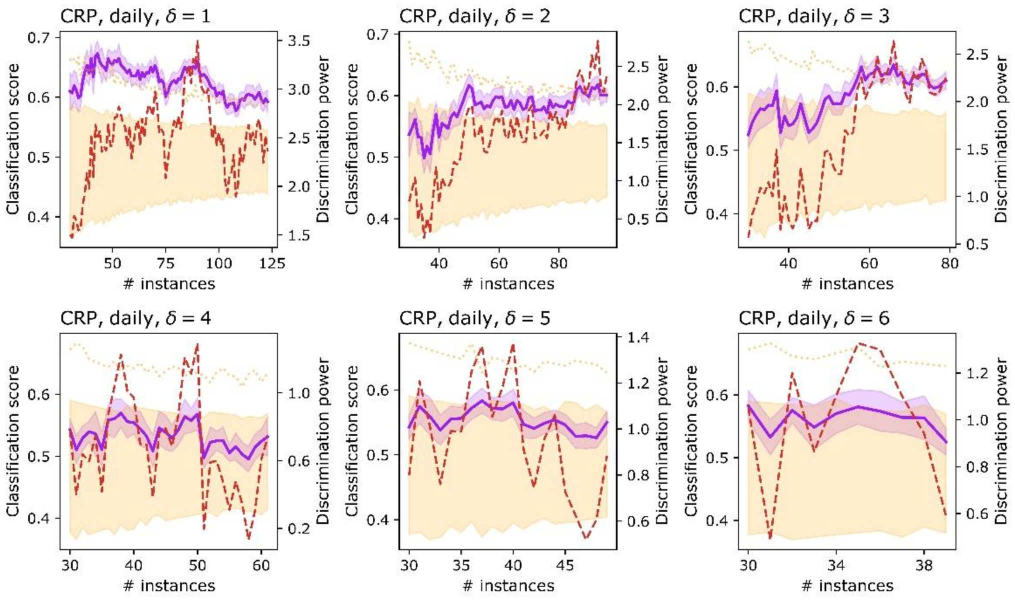

Figure S5. Classification score. Patients improving vs. deteriorating, as measured by the CRP level, as a function of the number of instances. Each panel represents a different number of days between the cytokine measurement and the assessment of the condition (δ). The purple lines represents the classification score (left Y axes), the red lines, the discrimination power (right Y axes).

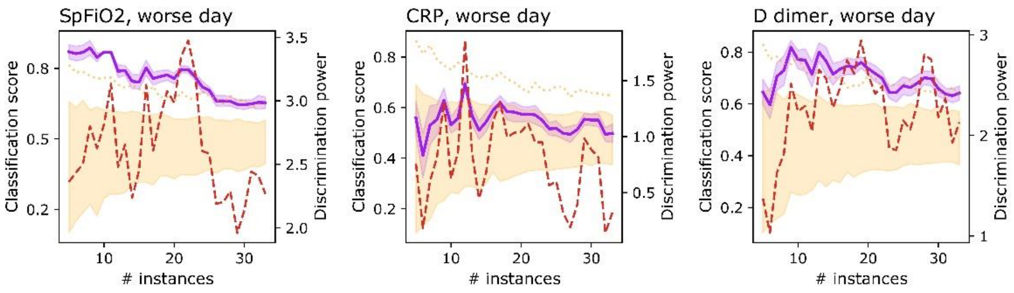

Figure S6. Classification score. Patient classification according to their condition on the worst day, as measured by the $SpFiO_2$ (left panel), CRP level (central panel), and D-dimer level (right panel), as a function of the number of instances.

Feature selection

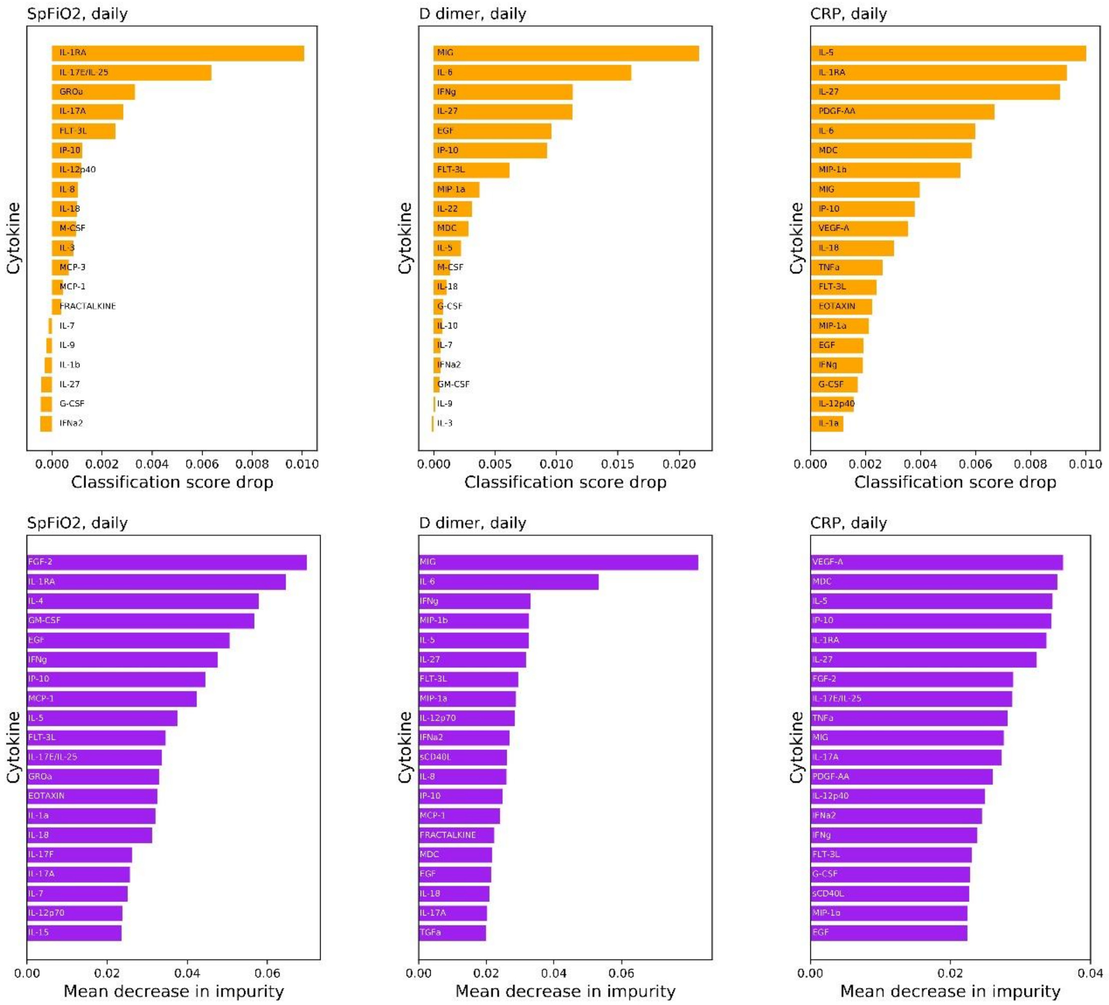

Figure S7. Feature selection. Ranking of the 20 most important cytokines, as considered by the RF classification model, according to the drop in classification score (top panels) and the mean decrease in impurity (bottom panels). Left, centre and right panels respectively correspond to models trying to predict the evolution of the patient condition as measured by SpFiO2, D dimer, and CRP
